# Supplementary material for: Cadherin clusters stabilized by a combination of specific and nonspecific cis-interactions
Source: eLife. 2020 Sep 2;9:e59035. doi: 10.7554/eLife.59035 (PMC7505656; doi:10.7554/eLife.59035)
Supplement: Supplementary file 1. [file elife-59035-supp1.docx]

**Supplementary File 1**

**Table a.** Number of trajectories and molecular observations used for diffusion analysis.

| **Experimental Condition** | **Total Trajectories** | **Trajectories Exhibiting FRET** | **Total Molecular Observations** |
| --- | --- | --- | --- |
| Wild-Type (~1,400 E-cad/µm^2^) | 4,603 | 961 | 103,595 |
| Wild-Type (~1,000 E-cad/µm^2^) | 4,628 | 799 | 102,196 |
| Mutant (~1,300 E-cad/µm^2^) | 2,157 | 523 | 49,693 |

**Table b.** All CCSDD Gaussian mixture model fitting parameters for the overall, low-FRET, and high-FRET CCSDDs for the one mutant and two wild-type E-cad experimental conditions. Numbers in the parentheses represent the uncertainties (standard deviation of 100 bootstrapped samples) in the least significant digit.

| **Experimental Condition** | **CCSDD** | **C_1_** | **C_2_** | **C_3_** | **D_1_ (µm^2^/s)** | **D_2_ (µm^2^/s)** | **D_3_ (µm^2^/s)** | **_short_ (µm^2^/s)** |
| --- | --- | --- | --- | --- | --- | --- | --- | --- |
| Wild-Type (~1,400 E-cad/µm^2^) | Overall | 0.30(4) | 0.58(3) | 0.11(1) | 0.13(1) | 0.39(2) | 1.53(8) | 0.444(3) |
|  | Low-FRET | 0.36(7) | 0.50(6) | 0.13(2) | 0.17(2) | 0.43(4) | 1.7(1) | 0.494(5) |
|  | High-FRET | 0.31(4) | 0.59(3) | 0.10(3) | 0.10(1) | 0.38(3) | 1.2(2) | 0.365(4) |
| Wild-Type (~1,000 E-cad/µm^2^) | Overall | 0.28(3) | 0.56(2) | 0.16(1) | 0.119(7) | 0.40(2) | 1.73(6) | 0.537(5) |
|  | Low-FRET | 0.27(3) | 0.57(2) | 0.17(1) | 0.129(8) | 0.40(2) | 1.76(6) | 0.556(5) |
|  | High-FRET | 0.40(5) | 0.48(3) | 0.12(3) | 0.10(1) | 0.43(5) | 1.7(2) | 0.44(1) |
| Mutant (~1,300 E-cad/µm^2^) | Overall | 0.34(6) | 0.54(4) | 0.12(1) | 0.16(1) | 0.46(4) | 1.9(1) | 0.531(7) |
|  | Low-FRET | 0.40(5) | 0.50(4) | 0.10(2) | 0.19(1) | 0.54(4) | 2.3(2) | 0.569(8) |
|  | High-FRET | 0.15(5) | 0.70(4) | 0.14(1) | 0.07(2) | 0.31(2) | 1.5(1) | 0.44(1) |

**Table c.** Protein solution concentrations for the two wild-type and one mutant E-cad conditions.

| **E-cad Used** | **Total Volume (µL)** | **Total Concentration (µM)** | **Unlabeled Concentration (µM)** | **Acceptor Concentration (nM)** | **Donor Concentration (pM)** |
| --- | --- | --- | --- | --- | --- |
| Wild-Type | 300 | 0.5 | 0.5 | 0.3 | 1 |
| Wild-Type | 300 | 0.3 | 0.3 | 0.3 | 1 |
| Mutant | 300 | 0.5 | 0.5 | 0.06 | 1 |

**Table d.** Calculated two-dimensional surface coverage values for the two wild-type conditions with high and intermediate protein concentrations and the one mutant condition with high protein concentration. Numbers in parentheses represent the uncertainties (standard error between 12 or 13 different movies) in the least significant digit.

| **Experimental Condition** | **Total Protein Solution Concentration (µM)** | **Surface Coverage (E-cad/µm^2^)** | **Fractional Surface Coverage** |
| --- | --- | --- | --- |
| Wild-Type | 0.5 | 1.37x10^3^(1) | 0.0124(1) |
| Wild-Type | 0.3 | 1.05x10^3^(2) | 0.0094(2) |
| Mutant | 0.5 | 1.26x10^3^(3) | 0.0113(2) |

**Table e.** Average transition rates between FRET states based on Markov model maximum likelihood estimated, beta-distributed transition probabilities. Numbers in parentheses represent the estimated uncertainties (square root of the Cramèr-Rao lower bound) in the least significant digit.

| **Experimental Condition** | **Average Transition Rate To High-FRET State (1/s)** | **Average Transition Rate To Low-FRET State (1/s)** |
| --- | --- | --- |
| Wild-Type (~1,400 E-cad/µm^2^) | 0.114(3) | 1.04(3) |
| Wild-Type (~1,000 E-cad/µm^2^) | 0.145(3) | 3.17(6) |
| Mutant (~1,300 E-cad/µm^2^) | 0.205(5) | 1.40(4) |

**Table f.** System details for concentration sensitivity analysis.

| **E-cad**  **Used** | **Number of Proteins** | **Surface Area**  **(nm^2^)** | **Concentration**  **(E-cad/µm^2^)** | **Number of**  **Simulation Runs** | **Average Cluster Size (# of E-cad)** |
| --- | --- | --- | --- | --- | --- |
| Wild-Type | 200 | 400 ×400 | 1,250 | 50 | 39.6 |
| Wild-Type | 100 | 400 ×400 | 625 | 50 | 26.0 |
| Wild-Type | 50 | 400 ×400 | 313 | 50 | 19.6 |
| Mutant | 200 | 400 ×400 | 1,250 | 50 | 28.4 |
| Mutant | 100 | 400 ×400 | 625 | 50 | 19.0 |
| Mutant | 50 | 400 ×400 | 313 | 50 | 9.0 |

**Table g.** System details for on/off rate sensitivity analysis.

| **E-cad**  **Used** | **Number of Proteins** | **Concentration**  **(E-cad/µm^2^)** | **Nonspecific Interaction On/off Rate**  **(s^-1^)** | **Specific Interaction On/off Rate**  **(s^-1^)** | **Number of**  **Simulation Runs** |
| --- | --- | --- | --- | --- | --- |
| Mutant | 50 | 1,250 | 2×10^6^/1×10^4^ | N/A | 10 |
| Mutant | 50 | 1,250 | 2×10^6^/1×10^3^ | N/A | 10 |
| Mutant | 50 | 1,250 | 2×10^6^/1×10^2^ | N/A | 10 |
| Mutant | 50 | 1,250 | 2×10^5^/1×10^4^ | N/A | 10 |
| Mutant | 50 | 1,250 | 2×10^5^/1×10^3^ | N/A | 10 |
| Mutant | 50 | 1,250 | 2×10^5^/1×10^2^ | N/A | 10 |
| Mutant | 50 | 1,250 | 2×10^4^/1×10^3^ | N/A | 10 |
| Mutant | 50 | 1,250 | 2×10^4^/1×10^2^ | N/A | 10 |
| Wild-Type | 50 | 1,250 | 2×10^5^/1×10^3^ | 1×10^8^/1×10^3^ | 20 |
| Wild-Type | 50 | 1,250 | 2×10^5^/1×10^3^ | 1×10^8^/1×10^2^ | 20 |
| Wild-Type | 50 | 1,250 | 2×10^5^/1×10^3^ | 1×10^8^/1×10 | 20 |
| Wild-Type | 50 | 1,250 | 2×10^5^/1×10^3^ | 1×10^7^/1×10^3^ | 10 |
| Wild-Type | 50 | 1,250 | 2×10^5^/1×10^3^ | 1×10^7^/1×10^2^ | 10 |
| Wild-Type | 50 | 1,250 | 2×10^5^/1×10^3^ | 1×10^7^/1×10 | 10 |
| Wild-Type | 50 | 1,250 | 2×10^5^/1×10^3^ | 1×10^6^/1×10^2^ | 10 |
| Wild-Type | 50 | 1,250 | 2×10^5^/1×10^3^ | 1×10^6^/1×10^2^ | 10 |
| Wild-Type | 50 | 1,250 | 2×10^5^/1×10^3^ | 1×10^6^/1×10 | 10 |

**Table h.** The rates used in kMC simulations that best fit the experimental cluster size distribution and experimental association time distributions.

|  | **On Rate (s^-1^****)** | **Off Rate (s^-1^****)** |
| --- | --- | --- |
| Nonspecific interaction | 2×10^5^ | 10^3^ |
| Specific interaction | 10^8^ | 10^2^ |
